# Population Genetic Structure of a Centipede Species with High Levels of Developmental Instability

**DOI:** 10.1371/journal.pone.0126245

**Published:** 2015-06-01

**Authors:** Giuseppe Fusco, Małgorzata Leśniewska, Leonardo Congiu, Giorgio Bertorelle

**Affiliations:** 1 Department of Biology, University of Padova, Padova, Italy; 2 Department of General Zoology, A. Mickiewicz University, Poznań, Poland; 3 Department of Life Sciences and Biotechnologies, University of Ferrara, Ferrara, Italy; Consiglio Nazionale delle Ricerche (CNR), ITALY

## Abstract

European populations of the geophilomorph centipede *Haplophilus subterraneus* show a high proportion of individuals with morphological anomalies, suggesting high levels of developmental instability. The broad geographic distribution of this phenomenon seems to exclude local environmental causes, but the source of instability is still to be identified. The goal of the present study was to collect quantitative data on the occurrence of phenodeviants in different populations, along with data on the patterns of genetic variation within and between populations, in order to investigate possible association between developmental instability and genetic features. In a sample of 11 populations of *H*. *subterraneus*, distributed in western and central Europe, we looked for phenodeviants, in particular with respect to trunk morphology, and studied genetic variation through the genotyping of microsatellite loci. Overall, no support was found to the idea that developmental instability in *H*. *subterraneus* is related to a specific patterns of genetic variation, including inbreeding estimates. We identified a major genetic partition that subdivides French populations from the others, and a low divergence among northwestern areas, which are possibly related to the post-glacial recolonization from southern refugia and/or to recent anthropogenic soil displacements. A weak correlation between individual number of leg bearing segments and the occurrence of trunk anomalies seems to support a trade-off between these two developmental traits. These results, complemented by preliminary data on developmental stability in two related species, suggest that the phenomenon has not a simple taxonomic distribution, while it exhibits an apparent localization in central and eastern Europe.

## Introduction

*Developmental stability* is the property of an organism to buffer random perturbations of the developmental process. The higher the level of developmental stability, the closer the features of the expressed phenotype to that of the *target phenotype*, i.e. the phenotype specified by the genetic makeup of the organism and the environmental conditions during its development [1–2]. The associated term *developmental instability* indicates a deficiency in resisting developmental disturbances [3].

The exact target phenotype is generally unknown, but body symmetries can be exploited to quantify developmental instability by measuring deviations from the expected symmetry. Random non heritable deviations from bilateral symmetry, or *fluctuating bilateral asymmetries* [4], have been largely employed to investigate levels and patterns of developmental instability in many taxa [5,6]. However, other types of body symmetry, such as those exhibited by organisms whose body architecture sees a repetition of body parts (e.g., segmented or radial organisms), can be used in addition or as an alternative to bilateral symmetry to implement an effective morphometric approach to the study of developmental instability [7]. In segmented animals, like annelids, arthropods, vertebrates and many other taxa [8], which present serially homologous structures along the main body axis, deviations from translational symmetry can be effectively analysed through morphometric analysis, either through the study of *fluctuating translational asymmetries* [7,9], or studying the occurrence of macroscopic defects at the level of serial structures (*frequency of segmental phenodeviants* [10]).

Developmental instability can have different causes, as for instance the individual genetic makeup at specific loci, the level of stochasticity of certain developmental processes and a variety of stressful environmental factors [5], and different causes can concur to the instability of the same developmental system [11].

The study of a Polish population of the geophilomorph centipede *Haplophilus subterraneus* (Shaw, 1794) (also recurring in literature with the synonym *Stigmatogaster subterranea*) revealed a high proportion of individuals with naturally occurring morphological anomalies of several kinds [12]. The specific morphology of these abnormalities, in relation to embryonic and early-postembryonic development of segmental structures [13–15], strongly suggests that trunk defects are congenital in these animals, and are thus indicators of high levels of developmental instability.

A preliminary survey on the geographical distribution of the phenomenon [16] showed that segmental anomalies of the same kind occur with comparable high frequency also in other four European populations of the same species, localized both within and outside the species’ natural range. The causes of such high levels of developmental disturbances are still to be identified, but its distribution in different areas seems to exclude local environmental factors (e.g., soil contamination by mutagenic agents).

In order to further investigate the occurrence of morphological anomalies in *H*. *subterraneus* at a wider geographical scale, we conducted a targeted collection campaign through central and western Europe. We collected quantitative data on the occurrence of phenodeviants in each population, and on the pattern of genetic variation within and between populations. A first inspection of the morphological data of this campaign, supplemented with data from other collections in central and western Europe, south Britain and Jutland confirmed the occurrence of high level of developmental instability for all European populations [17].

Here we analyse the geographic distribution of anomalies as indicators of developmental instability, examine the patterns of genetic variation as indicators of demographic events such as bottlenecks (and inbreeding), and study the possible association between morphological and genetic features of *H*. *subterraneus* individuals and population.

## Materials and Methods

### Study species

As most geophilomorph centipede species, *Haplophilus subterraneus* is an active predator inhabiting litter and soil, where it feeds on other invertebrates. *H*. *subterraneus* is distributed in the central and western Europe, from the Pyrenees to southern British Isles and western Germany. It usually occurs in natural woodland sites, but it is also commonly found in synanthropic sites like urban parks and cemeteries [18]. Isolated and possibly allochthonous populations are scattered close to human settlements in the remaining part of central and north-western Europe [19]. For further details on the species geographic record and habitat choice see [17].

### Sampling

**Fig 1:**
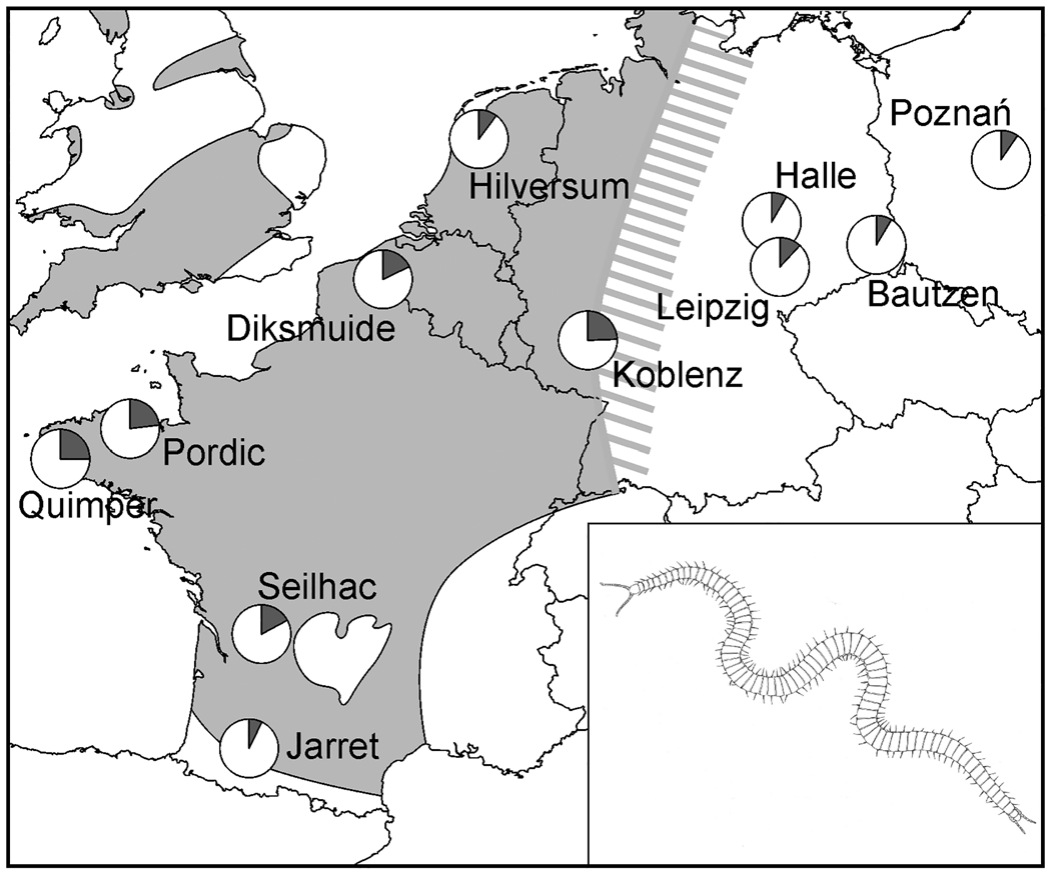
Geographic distribution of the eleven sampling sites for *H*. *subterraneus*. Pie charts report the relative frequencies of individual with normal phenotypes (white) and with trunk anomalies (dark gray). The light gray area is the natural range of the species after [19]. In the insert, the habitus of the species (body length about 60 mm).

The present study is based on a sample of 11 populations of *Haplophilus subterraneus*, distributed in western and central Europe, from southern France to central Poland, that is, across the whole natural range of the species and beyond its east border (Fig 1). Sampling sites include natural, semi-natural and synanthropic habitats (Table 1).

**Table 1:** Geographic location, habitat and sample sizes for the genetic and morphological analyses in the 11 sampled populations of *H*. *subterraneus*.

Population	Habitat	Sample size (genetics)^a^	Sample size (morphology)
Jarret (France)	deciduous forest	3	14
Seilhac (France)	mixed forest	20	35
Quimper (France)	park	19	32
Pordic (France)	deciduous forest	19	65
Diksmuide (Belgium)	park	20	28
Hilversum (The Netherlands)	arboretum	22	69
Koblenz (Germany)	cemetery	20	41
Leipzig (Germany)	park	17	42
Halle (Germany)	square	20	59
Bautzen (Germany)	cemetery	20	35
Poznań (Poland)	park	20	598
Total		200	1018

^a^Genetically typed individuals are a subsample of the set of animals used for morphometric analyses, with the exception of Poznań where the two samples do not overlap.

Field sampling was carried out during May 2010 for all the populations to the exclusion of the Poznań population, which was sampled in different seasons from May 2007 to June 2010. As a standard method for geophilomorphs, animals were collected by direct sampling from litter, soil, or under stones, loose bark of trees, logs and stumps [16]. *H*. *subterraneus* is not an endangered or protected species, and no permission for field collection was required in any sampling location.

During the May 2010 sampling, most specimens where immediately stored in 75% ethanol, the standard preserving solution for this arthropod group. However, approximately twenty individuals for each population (when possible) were fixed in 100% ethanol and within minutes cut transversally in two pieces: the head plus a few anterior trunk segments (about eight) were stored in 100% ethanol, while the rest of trunk was placed in 75% ethanol. The reason for this procedure is that the optimal solution for preserving material aimed at DNA extraction and purification, i.e. absolute ethanol, is unsuitable for preservation of material destined to an accurate morphological inspection, as this produces extended coiling and contractions in the body, followed by its stiffening. On the other hand, preliminary tests on DNA purifications from samples preserved in 75% ethanol showed a high level of DNA degradation, that was successfully prevented by the use of pure ethanol. For the Poznań population, genotyped and morphologically inspected individuals form to two distinct (non-overlapping) samples, and animals were stored in ethanol entire (not cut). Overall, 1018 specimens were used for morphometric analyses, including the search for morphological anomalies, while genetic analysis was carried out on a partial subsample of 200 specimens (Table 1).

For further details on sampling sites and animal collection see [17]. All specimens are preserved in M. Leśniewska’s collection (Department of General Zoology, A. Mickiewicz University, Poznań, Poland).

### Number of leg-bearing segments and sex

The trunk of geophilomorph centipedes comprises one anterior segment bearing a pair of poisonous maxillipedes (forcipular segment), a series of variable length of leg-bearing segments, and a terminal apodous ano-genital region of uncertain segmental composition, but reputed segmentally invariant across species, sexes and individuals [20,21]. Thus, in geophilomorphs, variation in the number of trunk segments is traditionally expressed in terms of variation in the number of leg-bearing segments. In our sample, this measure is available for 1005 specimens. As common among geophilomorphs [22], the modal number of leg-bearing segments in females is two segments higher than in males [17]. We therefore incremented by two the number of leg-bearing segments of each male in the sample to obtain a single morphometric variable (here called LBS) for joint analyses. LBS is available for 996 specimen, the intersection of the subset of specimens (n = 1005) with measured number of leg bearing segments and the subset of sex determined specimens (n = 1004).

### Morphological anomalies

All specimens were examined by ML with a stereoscopic light microscope up to 80x magnification. The aberrant morphologies recorded in the sample have been classified on the basis of Leśniewska et al.’s [12] taxonomy of anomalies.

**Fig 2:**
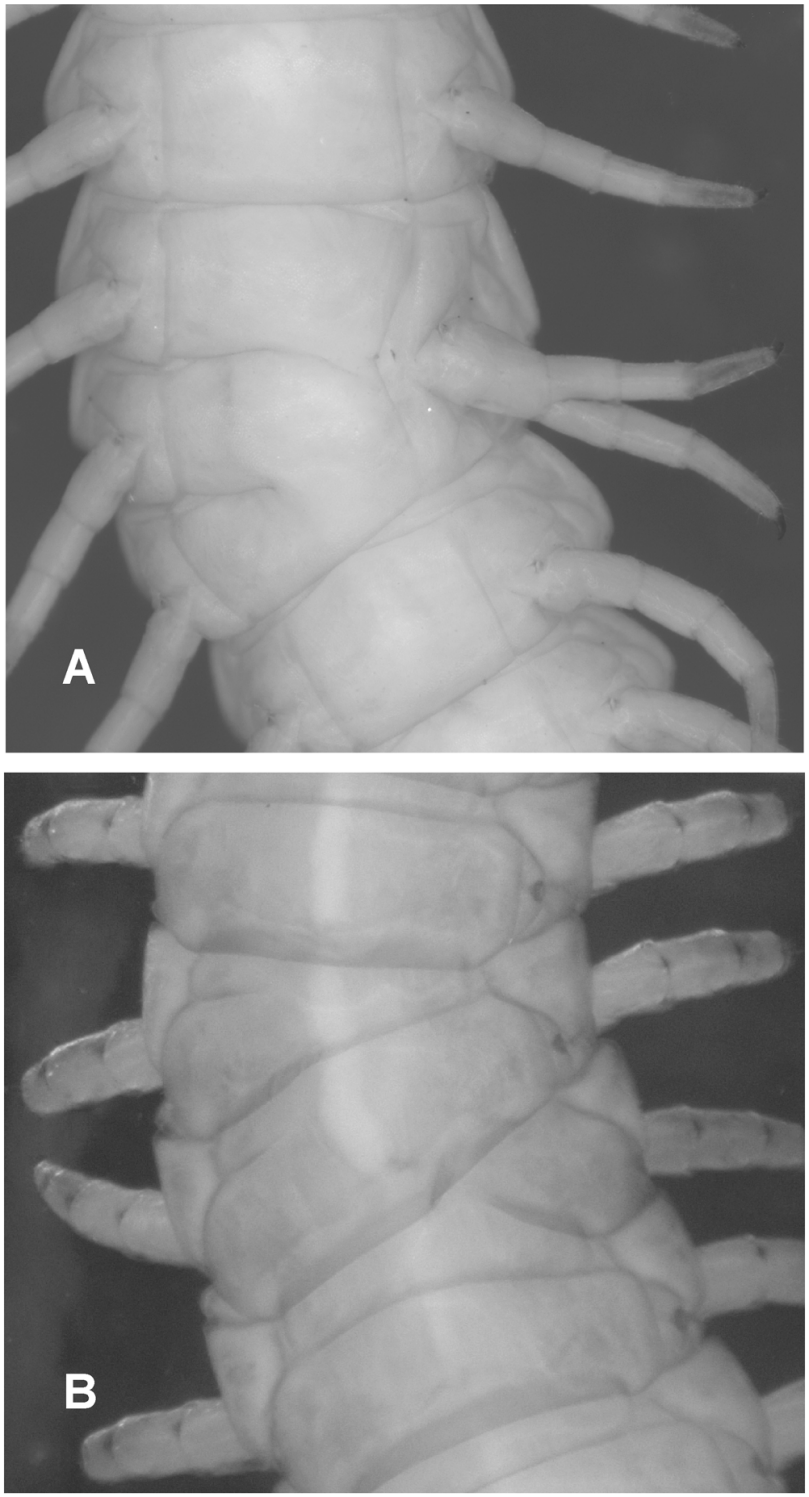
Example of trunk anomalies in two specimens of *H*. *subterraneus*. (**A**) Trunk shrinking (ventral view, anterior toward the top). The left side of the leg-bearing segments 75^th^-77^th^ is collapsed to the point that only one (bifurcated) leg emerges from the pleural region. (**B**) Dorsal mispairing (dorsal view, anterior toward the top). Dorsal sclerites of leg-bearing segments 19^th^-20^th^, rather than being transversally oriented within a given segment, present discordant left/right serial position (or identity).

Here we have considered separately trunk anomalies, affecting structures of the body to the exclusion of appendages, and appendages anomalies. In order to compare the frequency of morphological anomalies across populations, three categorical variables referable to single specimens were devised: i) a binary categorical variable that records the presence of trunk anomalies (*TA*, 0: absence, 1: presence); ii) a ranked categorical variable that gives a score (0–3) based on the ‘seriousness’ of trunk anomalies (*TAS*, 0: absence, 1: defects on sternal pore areas or virguliform fossae, 2: sclerite deformations, 3: dorsal mispairing or segment shrinking); iii) a binary categorical variable that records the presence of appendage anomalies (*AA*, 0: absence, 1: presence). Examples of morphological anomalies are reported in Fig 2.

### Genetic typing

Two-hundred specimens from 11 populations where genetically characterized. Genomic DNA was purified from head plus 6–8 trunk segments using a salting-out protocol [23].

Out of the nine microsatellite loci specifically isolated [24] and genotyped in all individuals, four loci (H.sub1, H.sub36, H.sub47, H.sub85) were cautiously excluded due to possible genotyping errors detected by the software MICRO-CHECKER 2.2.3 [25]. The other five loci (H.sub16, H.sub45, H.sub48, H.sub56, H.sub61) were used for data analyses. All experimental procedures from microsatellite amplification to scoring were performed following the instructions reported in the supporting information of [24], where also GenBank accession numbers can be found. In order to test the reproducibility of the genotyping, the analysis of the 20 individuals from the Poznań population was blind replicated in two different laboratories.

### Statistical analyses

#### Association between anomalies and non genetic variables

The relationships at the level of the individuals between the presence of anomalies (*TA* and *AA*: binary response variable; *TAS*: categorical response variable) and the population of origin, sex, and number of segments (two categorical and one count predictor variables) were analysed using a generalized linear model (with binomial or multinomial error terms) in two different data sets. The first data set included all available individuals (n≈1000, the exact number depending on the predictor), where more than half are from the Poznań population. The second data set (n≈480) included only a randomly selected subset of 60 (out of 598) individuals from Poznań, in order to have approximately similar sample sizes from each locality. Considering that the different sampling area could be also considered as a random extraction of European populations, we also tested the binary response variables *TA* and *AA* using the population of origin as a random effect in a generalized linear mixed model (as implemented in the R package lmerTest [26]).

#### Association between anomalies and genetic variables

The relationship at the level of the individuals between presence of anomalies (*TA* and *AA*: binary response variable; *TAS*: categorical variable) and genetic indicators of inbreeding (proportion of heterozygous loci and four different estimators of the inbreeding coefficient as implemented in Coancestry [27]: continuous predictors) was analysed in 180 individuals from 10 populations, using a generalized linear model (with binomial or multinomial error terms). The Poznań population could not be used in this analyses since morphological data for the genotyped individuals are missing.

The association between population variables related to developmental anomalies (frequencies of trunk and appendage anomalies) and genetic indices (average number of alleles, observed and expected heterozygosity, and inbreeding coefficient F_is_) was analysed by simple parametric (Pearson) and non parametric (Spearman) correlation analyses. Only ten points were available for these analyses, as the population of Jarret, with only three genotyped individuals, was not included.

#### Genetic variation and structure

Descriptive statistics of genetic diversity within populations were estimated in each population using Arlequin 3.5 [28]. The same software was used to test for deviation from Hardy—Weinberg equilibrium and for linkage disequilibrium between pairs of markers.

Genetic structure was analysed representing individual genotypes in a two dimensional space using the Factorial Correspondence Analysis (FCA), as implemented in the software Genetix 4.05 [29], and the Bayesian clustering method implemented in the software STRUCTURE 2.3.3 [30,31].

## Results

### Basic statistics

All the basic statistics elaborated from genetic and morphological data are reported in Table 2.

**Table 2:** Basic statistics for the sampled populations of *H*. *subterraneus*.

Population	TA	AA	TAS	LBS	H_e_	k	F_is_	Linkage	H-W
Jarret (France)	0.07	0.29	0.21	78.6	-	-	-	-	-
Seilhac (France)	0.17	0.34	0.34	80.8	0.32	2.4	0.15	1 (0–4)	0
Quimper (France)	0.25	0.25	0.44	81.6	0.57	4.2	0.09	2 (0–3; 3–4)	1 (4)
Pordic (France)	0.23	0.25	0.46	81.9	0.40	3.4	0.05	0	0
Diksmuide (Belgium)	0.18	0.11	0.39	82.8	0.50	3.2	-0.02	2 (0–1; 0–2)	0
Hilversum (The Netherlands)	0.10	0.26	0.16	81.2	0.33	2.6	0.08	1 (0–1)	1 (3)
Koblenz (Germany)	0.24	0.37	0.37	81.2	0.57	4.2	0.04	2 (1–2; 2–4)	1 (3)
Leipzig (Germany)	0.12	0.24	0.24	81.0	0.52	3.8	-0.08	1 (2–3)	0
Halle (Germany)	0.08	0.34	0.15	81.0	0.53	4.0	0.04	0	0
Bautzen (Germany)	0.09	0.17	0.20	81.3	0.58	4.0	-0.08	1 (0–1)	0
Poznań (Poland)	0.10	0.28	0.24	81.3	0.55	4.2	0.13*	1 (1–2)	0

Anomalies are reported as frequencies of trunk anomalies (TA), appendage anomalies (AA), and as the average trunk anomaly scores (TAS). LBS is the average number of leg-bearing segments corrected for sexual dimorphism (see text). H_e_ and k are the expected heterozygosity and the number of alleles, respectively (not reported for the Jarret population due to very limited number of genetically typed individuals). Fis = inbreeding coefficient (the asterisk indicates a significant deviation from zero with P<0.05). The last two columns report the number of significant (P<0.05) pairwise deviations from linkage equilibrium (pairs of loci in parenthesis), and the number of loci significantly (P<0.05) deviating from HW equilibrium (locus in parenthesis).

There are significant differences between populations for LBS (ANOVA, F = 11.51, P<0.0001, n = 996), with the populations of Jarret and Diksmuide presenting significantly extreme values (78.6 and 82.8, respectively).

The frequency of trunk phenodeviants varies between 7% and 25%, while the frequency of appendage phenodeviants varies between 11% and 37%. As expected, being based on the same kind of anomalies, *TA* and *TAS* means (the former corresponding simply to the population frequency, and the latter to the score average) are strongly correlated (Pearson, r = 0.924, P<0.0001, n = 11; Spearman’s rank, r = 0.8636, P = 0.0063, n = 11).

Most of the populations show a relatively high level of genetic variation, with expected heterozygosity larger than 50%. Lower values are observed in France (Seilhac: 0.32; Pordic: 0.40) and the Netherlands (0.33). These three populations show also slightly lower number of alleles. The inbreeding coefficient F_is_ is significantly larger than 0 only in the city park of Poznań.

### Genetic structure

**Fig 3:**
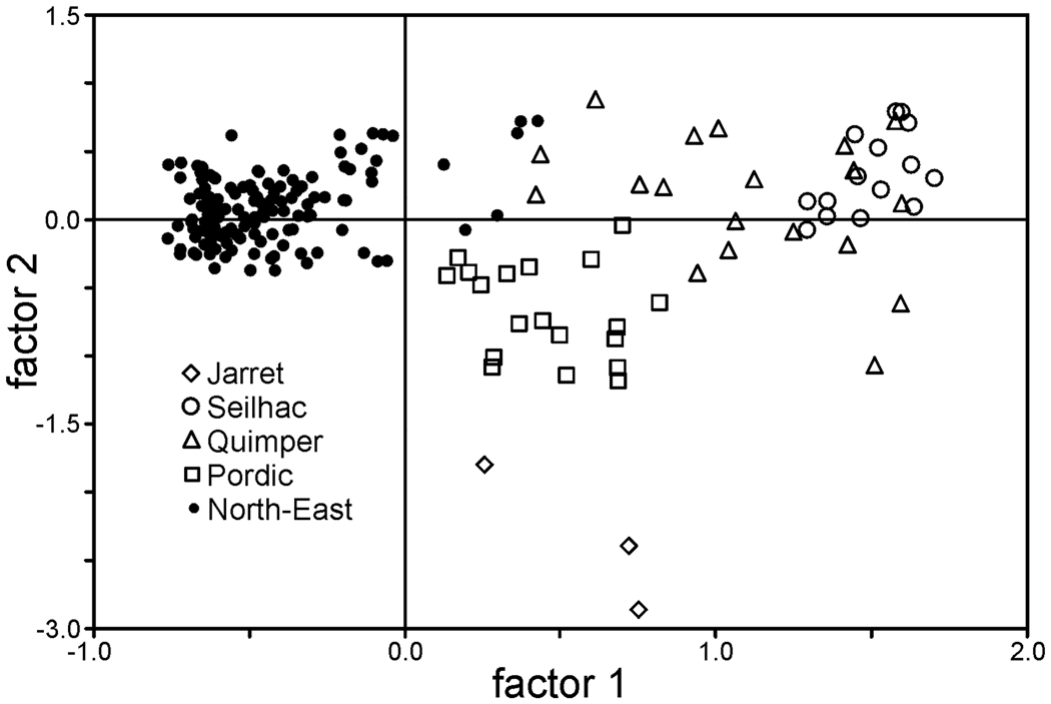
Bi-dimensional representation of individual genotypes provided by the factorial correspondence analysis. Each allele is used as an independent variable, with no a priori assumptions about grouping. Individuals of North-Eastern populations are represented by the same symbol as they intermix in the plot.

Significant and high genetic structure is observed across the whole distribution range, with global F_st_ = 0.29 (P<0.01, S1 Table). The graphical representation of the individual genotypes (FCA, Fig 3) suggest that the major genetic partition is between the populations in France and all the others. In fact, only few individuals from the North-Eastern areas (all from Koblenz) cluster with the left cloud of French individuals. In addition, whereas North-Eastern individuals form largely overlapping clouds (not shown in Fig 3 to reduce the number of symbols), the individuals from at least three French populations (Pordic, Seilhac, and Jarret) are more genetically structured and can be clearly assigned to their population of origin. The Bayesian clustering analysis (STRUCTURE) supports the FCA plot when the number of groups was set to two, with France and North-Eastern populations clearly affiliated to one of each group (S1 Fig).

### Association analyses

Significant association between inbreeding and the presence/absence of anomalies were not identified, independently on the inbreeding estimator used and the kind and severity of the morphological anomaly. On the contrary, the population of origin, the number of segments, and their interaction had significant effects on *TA* when the complete data set was used (likelihood tests: P = 0.002, P = 0.02, and P = 0.0003, respectively; n = 996). When *TAS* is analysed, only the effects of the population of origin and its interaction with the number of segment remain significant (P = 0.0003 and P = 0.005, respectively; n = 996). No significant effect of these predictor variables on *AA* was recorded. Analysing the reduced and more balanced data set, the interaction between population and number of segments was significant both in the analysis of *TA* and *TAS* (P = 0.001 and P = 0.007, respectively; n = 464), and the effect of the population of origin was significant only for *TAS* (P = 0.009). Again, *AA* did not show any significant response. Considering the population of origin as a random effect in a linear generalized mixed model, the population of origin and the interaction between population of origin and number of segment had significant effects on *TA* both in the complete data set (likelihood tests: P = 0.009 and P = 0.011, respectively) and in the reduced data set (likelihood tests: P = 0.002 and P = 0.003, respectively). No significant effect on *AA* was recorded.

Both parametric and non-parametric correlations between population frequencies of anomalies and all measures of genetic variation (H_e_, k and F_is_) produced non-significant results.

## Discussion

### Developmental instability and genetic variation

Overall, our investigation on the geographic distribution of the occurrence of anomalies, complemented by the check for correlations between the incidence of anomalies and genetic variation patterns, provide no support to the idea that developmental instability recorded in the European populations of the centipede *H*. *subterraneus* is related to some specific genetic feature of the populations or the single individuals. In particular, we did not find any association between low genetic variation and the frequency of anomalies, or estimated inbreeding coefficients and presence/absence of anomalies in single individuals. Similarly, no apparent relationship between genetic divergence and the frequency of anomalies was observed. Our data seems therefore to exclude that inbreeding depression (possibly related to reduced population size) or outbreeding depression (possibly related to individual displacements due to human activities) are the cause of the morphological defects observed in this centipede.

Unlike the variables of trunk anomalies (*TA* and *TAS*), the means of the variable of appendage anomalies (*AA*) do not differ significantly among populations. This adds to other arguments on the questionable value of appendage anomalies as indicators of developmental stability. In fact, while trunk anomalies are likely congenital, the scarce information on the regenerative potential and the morphology of healing in geophilomorph appendages [32,33] does not allow to confidently discriminate between appendage congenital defects and defects resulting from imperfect regeneration in these structures. For these reasons, frequency data of appendage anomalies, which should be taken with reserve [16], are not discussed further.

### Geographic patterns

The ecology of the species, in combination with its current geographic distribution, suggests a post-glacial history of expansion from some refuge areas, possibly on the northern side of Pyrenees. This area is one of the so the called ‘northern refuges’ in Europe (with respect to the more widely recognized refuge areas in the Mediterranean peninsulas [34]), which apparently harboured several temperate species from the severe climatic conditions of the last glaciations [35]. *H*. *subterrraneus* would have subsequently colonized central and eastern Europe areas following a north-eastern route.

As observed in many other species [36–38], the present-day pattern of genetic variation observed in *H*. *subterrraneus* seems to reflect demographic changes due to the glaciation events. In fact, the much higher level of genetic structure observed in France compared to the northeastern European populations, and the major genetic partition of the samples in southern and northern populations, can be related to the fact that southern samples come from the more stable distribution range of this species, continuously occupied also during the glaciations due to favourable climatic conditions, whereas northern populations reflects a more recent history of post-glacial colonization accompanied by a genetic divergence from the refuge(s).

An alternative, or complementary, explanation for the lower structure in northern areas is that human displacement of garden soils in recent years (<200 years ago) prevented population differentiation. This hypothesis, compatible with the synanthropic habits of the species, could also explain why, on the average, northern populations have similar or slightly higher heterozygosities compared to the French groups, which is in general not expected in recolonized areas compared to refugia [39]. Passive transport with garden soil cannot however represent the exclusive explanation for the observed pattern of variation.

### Developmental instability additional patterns

The repetition of serially homologous structures along the main body axis is a common form of modularity, found in the body architecture of many multicellular eukaryote taxa. Several studies have explored how natural selection may have promoted the evolution of a modular body organization, and the potential of repetitive body units in taxa diversification [40]. However, comparably less attention has been paid to the possible influence of the developmental features of modularity on the evolvability of this primary morphological trait.

We found a significant, although not strong, positive correlation between individual number of leg bearing segments and the occurrence of trunk anomalies in *H*. *subterraneus*. This correlation concurs with the observation that most of the teratological cases so far recorded in the whole Geophilomorpha are found in the taxon Himantariidae [12], characterized by very long and polypodous species, to which *H*. *subterraneus* belongs. These observations might suggest the idea of a trade-off between the number of body modules and developmental stability, as if the length of a segmental series, in addition or in combination with other factors, could affect the developmental precision of its phenotypic expression. This may seem at odds with the geophilomorph relatively homonomous (i.e., morphologically scarcely dedifferentiated) trunk segmental pattern, as a relatively simple morphological design can be supposed to be associated to equally simple developmental processes of segmental patterning and differentiation. However, measures of morphological complexity of the trunk segmental structures [9], mapped onto the comparative background of Chilopoda phylogeny [41], show that the segmental pattern of geophilomorphs is a derived state with respect to the plesiomorphic oligomerous and more heteronomous condition observed in more basal Chilopoda, like for instance the lithobiomorphs. Thus, the relatively simple trunk segmental pattern of geophilomorphs might nonetheless involve a comparatively complex developmental control.

However specifically aimed studies, extended to other segmented (or modular) taxa, are needed to assesses effectiveness and/or generality of this possible trade-off.

Finally, we note that the taxonomic boundaries of this phenomenon cannot easily be defined. Similarly to all populations of *H*. *subterraneus* sufficiently sampled and reasonably well inspected, which show high frequencies of abnormal morphologies, a comparably high frequency of abnormalities characterizes also a population of the closely related species *H*. *souletinus*, from the Pyrenees [17]. However, the inspection of 115 specimens of another closely related species, *Stigmatogaster gracilis*, from Italy, revealed only appendage anomalies (in 29.6% of the specimens), but no one trunk anomaly. Because of the doubtful value of appendage anomalies as indicators of developmental stability (see above), there is therefore no compelling evidence of high levels of developmental instability in this species. This result is noteworthy, as the most recent geophilomorph phylogeny [42] resolves *S*. *gracilis* as the sister species of *H*. *subterraneus*, with *H*. *souletinus* branching out basally with respect the other two species. Thus, the phenomenon seems to have a taxonomic distribution that cannot be circumscribed to a single clade, while, irrespective of the phylogeny, although not caused by strictly local factors, it presents an apparent localization in central and eastern Europe. Further studies will be necessary to understand the causes of this developmental instability and its taxonomic and geographic patterns.

## Supporting Information

S1 Dataset Raw data used for morphological and genetic analyses. (XLS)

S1 Fig *Structure* bar plot of the genetic compositions of single individuals. (PDF)

S1 Table Pairwise differentiation (F_st_) between population. (PDF)
