# Supplementary material for: Population Genetic Structure of a Centipede Species with High Levels of Developmental Instability
Source: PLoS One. 2015 Jun 1;10(6):e0126245. doi: 10.1371/journal.pone.0126245 (PMC4452494; doi:10.1371/journal.pone.0126245)

Supporting information

Population genetic structure of a centipede species with high levels of developmental instability

Fusco G., Leśniewska M., Congiu L. and Bertorelle G.

Figure S1. Bar plot representing the genetic composition of single individuals (thin vertical columns) as inferred from STRUCTURE with K=2. We applied the LOCPRIOR with admixture model, and the assumption of correlated allele frequencies among populations. The burn-in period was set to 50000, and the run consisted of 10^7 iterations.

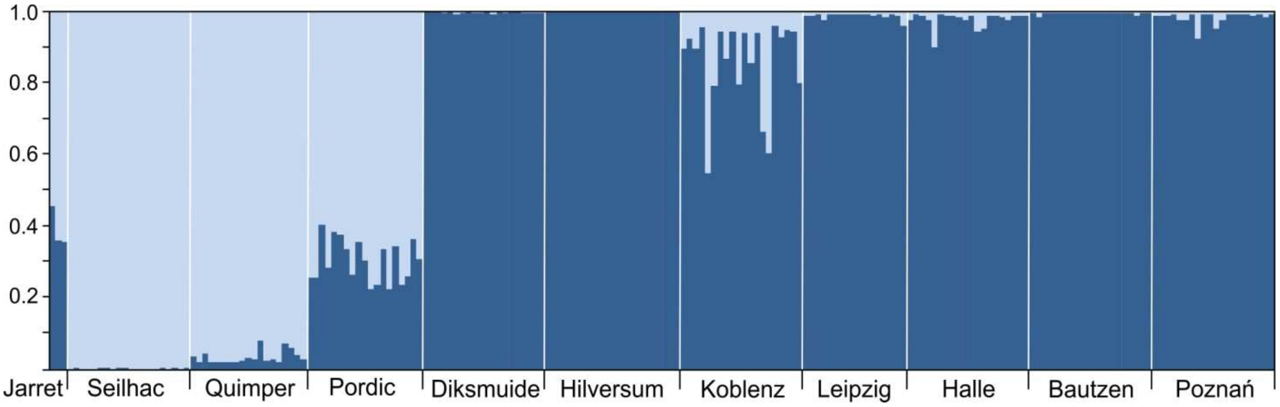

Supplement: S1 Fig — (PDF) [file pone.0126245.s002.pdf]
