# Supplementary material for: Population Genetic Structure of a Centipede Species with High Levels of Developmental Instability
Source: PLoS One. 2015 Jun 1;10(6):e0126245. doi: 10.1371/journal.pone.0126245 (PMC4452494; doi:10.1371/journal.pone.0126245)
Supplement: S1 Table — (PDF) [file pone.0126245.s003.pdf]

Supporting information

Population genetic structure of a centipede species with high levels of developmental instability

Fusco G., Leśniewska M., Congiu L. and Bertorelle G.

Table S1. Pairwise differentiation (Fst) between populations. Underlined values are not significantly different from zero after Bonferroni correction (P>0.0009). The value in bold is not significant with P>0.05.

	Jarret	Seilhac	Quimper	Pordic	Diksm.	Hilvers.	Koblenz	Leipzig	Halle	Bautzen
Seilhac	0.559									
Quimper	0.404	0.238								
Pordic	0.366	0.530	0.302							
Diksmuide	0.467	0.576	0.333	0.342						
Hilversum	0.534	0.687	0.452	0.429	0.083					
Koblenz	0.427	0.495	0.222	0.308	0.053	0.131				
Leipzig	0.426	0.556	0.298	0.353	0.106	0.171	0.060			
Halle	0.432	0.571	0.308	0.321	0.098	0.095	0.060	0.059		
Bautzen	0.376	0.543	0.294	0.307	0.054	0.090	0.047	0.039	**0.008**	
Poznań	0.396	0.552	0.321	0.305	0.033	0.063	0.053	0.081	0.046	0.024
